# Quality of National Disease Surveillance Reporting before and during COVID-19: A Mixed-Method Study in Indonesia

**DOI:** 10.3390/ijerph19052728

**Published:** 2022-02-26

**Authors:** Muhammad Hardhantyo, Hanevi Djasri, Aldilas Achmad Nursetyo, Andriani Yulianti, Bernadeta Rachela Adipradipta, William Hawley, Jennifer Mika, Catharina Yekti Praptiningsih, Amalya Mangiri, Endang Burni Prasetyowati, Laura Brye

**Affiliations:** 1 Center for Health Policy and Management, Faculty of Medicine, Public Health and Nursing, Universitas Gadjah Mada, Yogyakarta 55281, Indonesia; hanevi.djasri@ugm.ac.id (H.D.); mail.aldilas@gmail.com (A.A.N.); ndiani_86@yahoo.com (A.Y.); bernadeta.rachela@gmail.com (B.R.A.); 2 Faculty of Health Science, Universitas Respati Yogyakarta, Yogyakarta 55281, Indonesia; 3 Centers for Disease Control and Prevention, Division of Global Health Protection, Atlanta, GA 30329, USA; byh0@cdc.gov (W.H.); ziz5@cdc.gov (J.M.); xel3@cdc.gov (C.Y.P.); xel5@cdc.gov (A.M.); 4 Directorate of Surveillance and Health Quarantine, Ministry of Health, Jakarta 12950, Indonesia; aya_fhya@yahoo.com; 5 Project HOPE, Bethesda, MD 20814, USA; lbrye@projecthope.org

**Keywords:** COVID-19, public health measures, health care system, disease burden, public health resources

## Abstract

Background: Global COVID-19 outbreaks in early 2020 have burdened health workers, among them surveillance workers who have the responsibility to undertake routine disease surveillance activities. The aim of this study was to describe the quality of the implementation of Indonesia’s Early Warning and Response Alert System (EWARS) for disease surveillance and to measure the burden of disease surveillance reporting quality before and during the COVID-19 epidemic in Indonesia. Methods: A mixed-method approach was used. A total of 38 informants from regional health offices participated in Focus Group Discussion (FGD) and In-Depth Interview (IDI) for informants from Ministry of Health. The FGD and IDI were conducted using online video communication. Yearly completeness and timeliness of reporting of 34 provinces were collected from the application. Qualitative data were analyzed thematically, and quantitative data were analyzed descriptively. Results: Major implementation gaps were found in poorly distributed human resources and regional infrastructure inequity. National reporting from 2017–2019 showed an increasing trend of completeness (55%, 64%, and 75%, respectively) and timeliness (55%, 64%, and 75%, respectively). However, the quality of the reporting dropped to 53% and 34% in 2020 concomitant with the SARS-CoV2 epidemic. Conclusions: Report completeness and timeliness are likely related to regional infrastructure inequity and the COVID-19 epidemic. It is recommended to increase report capacities with an automatic EWARS application linked systems in hospitals and laboratories.

## 1. Introduction

In late 2019, newly found acute respiratory disease caused by the novel coronavirus SARS-CoV-2 began to spread in Wuhan, China. Later the disease, then called COVID-19, started spreading to adjacent countries and multiple continents. In March 2020, the World Health Organization (WHO) declared the COVID-19 outbreak a global pandemic. By January 2022, more than 340 million human cases and 5.5 million deaths due to COVID-19 were reported [1].

Recent globalization and urbanization, increasing human–animal contact, and health worker shortages are some of the underlying causes of the pandemic and are becoming more likely. Consequently, every country has a high risk of becoming a *hotspot* for a disease outbreak with the potential to cause a global pandemic. Pandemics can affect any country, regardless of location or socioeconomic status [2]. The spread of pandemics is closely related to a region/country’s readiness and ability to mitigate disease outbreaks that have a pandemic potential [3].

According to the International Health Regulation State Party Annual Reporting (IHR SPAR) findings across 182 countries, many countries are not ready to deal with the next pandemic. This readiness is measured by five aspects: (i) prevention, (ii) detection, (iii) response, (iv) availability of supporting facilities, and (v) operational readiness. One crucial factor used to assess the readiness of a region/country in dealing with a pandemic is the availability of adequate health data and information [4]. Such data are crucial for healthcare provision and government decision making during the crisis. The importance of transparent health data before and during a pandemic augments the readiness of the government and other stakeholders to develop science-based approaches to control disease outbreaks [5,6].

The World Health Organization (WHO) requires that every country can detect, assess, report, and respond to public health emergencies of international concern (PHEIC) [7,8]. The WHO Benchmark for surveillance states that strengthened surveillance systems can detect events of significance for public health and health security, improve communication and collaboration across sectors, and between subnational (local and intermediate), national and international levels of authority regarding surveillance of events of public health significance. The benchmark also aims to improve national and intermediate level regional capacity to analyze data, strengthen early warning surveillance, including interoperable, interconnected electronic tools. This would incorporate epidemiological, clinical, laboratory, environmental testing, and product safety and quality of bioinformatics data, and advances in fulfilling core capacity requirements for surveillance according to the IHR [8,9]. For this purpose, WHO proposed a system called Early Warning and Response System. The framework recommends that the disease surveillance system should build electronically and integrated with response system. It allows the national health system to deliver timely responses to the indication of disease outbreaks [10].

To accomplish those standards, the Indonesian Ministry of Health (MoH) developed an electronic Early Warning Alert and Response System (EWARS) program in 2009 in collaboration with the World Health Organization (WHO). The EWARS application records weekly surveillance data for 23 diseases in Indonesia. However, the current application can only receive data from public health centers and has been integrated data from private, public, or clinic providers [11]. Currently, EWARS uses indicator-based or event-based surveillance systems that rely upon reports from officials to detect disease, conditions, and events. As part of efforts to increase the sensitivity of the surveillance system, an information technology application can be built to facilitate immediate reporting of alerts that can indicate the possibility of a serious public health threat [12,13]. Some surveillance systems apply alert methods based on timeliness (the duration between the first true alarm and the onset of the outbreak) threshold which is developed from epidemiological time series analysis [14]. For example, the Chinese Information Platform for Disease Control and Prevention performs automatic analysis and calculation of nationwide notifiable infectious disease surveillance data by leveraging different early warning algorithms for different diseases, and sends timely signals on detected abnormal case increase or clustering to local county/district CDCs via short message service (SMS) [15,16].

There have been several studies evaluating national EWARS in several countries. Keita et al. evaluated EWARS in Democratic Republic of Congo specifically built for Ebola virus disease. The study quantitatively assessed the effectiveness of EWARS as a tool to prevent further spreading of the disease [17]. A study on Zanzibar, Tanzania’s EWARS application assessed the capacity for early detection and response of infectious disease using a mixed-method study design [18]. Evaluations were also conducted in Uganda, Liberia, and Malawi on the Integrated Disease Surveillance and Response (IDSR) strategies. Findings from these studies concluded inadequate numbers of trained staff, inadequate funding, irregular supervision and high turnover of trained staff [19,20,21]. The earliest publication on Indonesia EWARS evaluation by Hapsari et al. described the first version of EWARS on 2009–2011 in six provinces. The study assessed the use of EWARS and laboratory capacity at the provincial level. The study concluded that the alert monitoring by using EWARS could be used as an evaluation tool to measure the quality of response conducted by local health officers or Rapid Response Team (RRT). Despite the lack of laboratory confirmation and the alerts detected by the system, EWARS was well accepted in the provinces [22]. The other study published by Manurung evaluated EWARS implementation in Papua, the easternmost region in Indonesia, using a qualitative study design. The results of the study showed increased disease control coordination among health jurisdictional levels in the province and the barriers to complete reporting and response to alerts included limited human and funding resources for surveillance, lack of epidemiological training, and technical limitations imposed by limited internet and mobile communication infrastructure in this remote region [23]. However, there is no study to evaluate the system nationally and investigate the underlying problem in Indonesia in specific situations such as the current COVID-19 pandemic.

This study aims to evaluate the quality of implementation of Indonesia’s EWARS for disease surveillance, to determine whether COVID-19 affects the data reporting quality before and during the COVID-19 epidemic in Indonesia.

## 2. Materials and Methods

### 2.1. Study Objectives and Design

We further address the following questions in this study: (a) What is the quality of Indonesia’s Early Warning and Response Alert System (EWARS)? and (b) Does COVID-19 put more burden in disease surveillance reporting using EWARS compared to previous years before the pandemic?

The methods of qualitative research and descriptive quantitative data have proven useful in the evaluation of surveillance activities in pandemic preparedness [24,25]. Thus, we use a mix of quantitative and qualitative methods to identify the implementation gaps of EWARS in Indonesia based on the timeliness and completeness of reporting of diseases.

The research process is divided into several stages. The first stage is the quantitative stage where we analyze yearly completeness and timeliness at national and regional level from 2017 to 2020. The second stage is the qualitative stage where we measure the challenges and progress of implementing the EWARS system.

### 2.2. Study Setting and Participants

**Figure 1:**
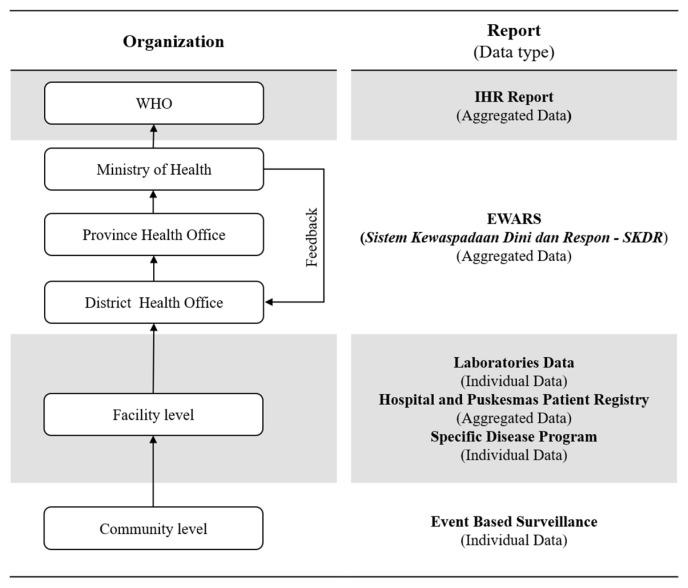
Flow chart of data report and management in the Indonesian Early Warning Alert and System (EWARS).

Indonesia first developed their EWARS application on 2009 called Sistem Kewaspadaan Dini dan Respon (SKDR). The application has been tested in six different provinces in the west and central regions [22]. Currently, the application facilitates weekly bottom-up indicator-based reporting (IBS) from primary healthcare centers (PHCs) on potentially outbreaks of infectious diseases. The diseases include dengue fever, diarrhea, influenza-like illness, pneumonia, dysentery, typhoid, and polio, and the other infectious diseases comprise 23 different diseases. Another feature has been added, which is event-based surveillance (EBS) reporting where PHC report immediately if there is any highly dangerous and infectious disease such as Ebola virus disease and malaria [26]. Figure 1 shows the flow chart of the data reporting and management of the Early Warning EWARS in Indonesia. Ministry of Health (MoH), through their subordinate administrative levels, the Province Health Office (PHO) and District Health Office (DHO), is responsible for verifying and coordinating the response, including epidemiological investigations by 10,134 PHCs [8].

The first stage is quantitative stage will divide the focus of study into six sub-regions of Indonesia: Sumatra, Jawa, Bali, Kalimantan, Sulawesi, Nusa Tenggara, and Maluku and Papua [27]. The qualitative stage included purposive sampling based on regions East Kalimantan, Yogyakarta, East Nusa Tenggara, and Papua. The consideration of this selection ensures participants are from diverse regions of Indonesia. Yogyakarta represents western part, East Kalimantan represents the central part, while East Nusa Tenggara and Papua represent the eastern part of Indonesia. The participants of this study represent EWARS managers at the national and regional level. The total participants in this qualitative stage are 41 participants. The participants of the focus group discussion (FGD) were invited through a letter sent to their institution. Participants were not known to the interviewers prior to the interview. Informed consent was obtained from all participants at the time of scheduling the interview or discussion.

### 2.3. Measures Quality of Indonesia’s EWARS 

Several studies are using timeliness and completeness as key indicators to measure the quality of their national EWARS [20]. In evaluating EWARS, it is important to evaluate the human resources, national and regional organization, and also the capacity to detect cases based on the availability of laboratories [28,29]. In this study, we follow evaluation guidelines developed by Centers for Disease Control and Prevention (CDC) using timeliness and completeness as key indicators. The framework is organized into four categories: system description, outbreak detection, implementation challenges, and improvement strategies to better understand the surveillance approaches [30]. Moreover, the study included the COVID-19 pandemic and its impact on the EWARS performance.

#### 2.3.1. Quantitative Stage (Quality of Indonesia’s EWARS)

Timeliness and completeness of EWARS reports were extracted from 34 provinces and 514 districts. Data were collected from information published in the public domain of Indonesian EWARS reports http://skdr.surveilans.org/home/lengkap/ for completeness and http://skdr.surveilans.org/home/tepat/ for timeliness from 2017–2020 accessed on 25 January 2021. According to Ministry of Health Act No 45 of 2014, report completeness was defined as the proportion of reports completed per expected reports from health facilities. The system in EWARS automatically calculates proportions of completeness and timeliness. Timeliness is the proportion of health facilities that submit their report on time every week. EWARS reports were considered “on time” if submitted into the system during the following week. A minimum quality of standard completeness and timeliness of 90% is required by Government of Indonesia (GoI). In this study, we use the outcome of the percentage of districts with a complete and “on-time” report. For completeness, districts with the minimum completeness report of 90% were coded with 1 = complete and 0 = incomplete, while for timeliness outcome, districts with the minimum of 90% “on time” report were coded with 1 = “on-time”, and 0 = “late report” for the districts which submitted the report later than the due date of the following week. Completeness and timeliness were measured in average percentages and stratified by region.

#### 2.3.2. Qualitative Stage (Burden of Disease Surveillance during COVID-19 Pandemic)

In the qualitative stage, the data were collected through semi-structured focused group discussions with a representative from surveillance personnel at national level and in four sub-national regions. For study instrument, we developed an interview guideline to evaluate the current EWARS system (Supplementary Materials). We applied *Updated guidelines for evaluating public health surveillance systems* published by the CDC [30]. The interview guidelines were structured to collect demographic profiles of the respondents, their knowledge and awareness on the implementation and obstacles of the EWARS surveillance system, and areas for improvement, such as processes related to data collection, reporting practices, challenges in interoperability, or human resources in the selected provinces. 

To validate the study instrument, we ask experts in public health and human health resources (HHR) field to review the study instrument. These experts have extensive experience in conducting studies on the quality of national program implementation. Each item on the questionnaire was reviewed by experts to ensure that it was relevant. In order to ensure that participants fully comprehended the questionnaire, several colleagues were requested to review the questionnaires and provide feedback.

Due to the strict regulation during the COVID-19 pandemic, all FGDs were performed online via video conferencing software. Interviews of around 60 min were conducted in Bahasa Indonesia and digitally recorded for transcribing. The FGDs were conducted during January 2021.

### 2.4. Data Analysis

Data were analyzed using a calculated mean and standard deviation of the proportion for the national regional level stratified by each year from 2017 to 2020. Districts with 90% completeness and timeliness were calculated as proportion percentage at their respective province level with standard deviation and stratified by the year from 2017 to 2020. Before further analysis, the study tested the normality of data distribution using the Shapiro–Wilk test and turnout the data was skewed with *p* < 0.05 significance level. To measure the differences between years, the study used the Kruskal–Wallis test and Pairwise Wilcoxon Signed Rank test. A significance level of *p* ≤ 0.05 was used.

This study also used a thematic analysis by applying CDC’s framework for evaluating surveillance systems for early detection of outbreaks [30]. We present a description of current EWARS activities, including the system description, outbreak detection, implementation challenges, and improvement strategies obtained from the participants. Transcripts were analyzed and coded manually. Major themes developed through a content analysis were constructed and integrated with descriptive analysis.

### 2.5. Ethical Approval

The study protocol ethical approval was received from the Medical and Health Research Ethics Committee of the Faculty of Medicine Public Health and Nursing, Universitas Gadjah Mada, Indonesia with KE/FK/0011/EC/2021 on 12 January 2021.

## 3. Results

To describe the quality of EWARS implementation, we arrange the result section into following. First, we describe the result of quantitative analysis of EWARS report showing rate of completeness and timeliness. The analysis included differences of performance from time to time (2017–2020). Second, we describe the qualitative analysis result based on FGD with the officials in each Indonesia region. This section will show quotes relevant with the topics from the interview guideline.

### 3.1. Quantitative Stage (Quality of Indonesia’s EWARS)

**Figure 2:**
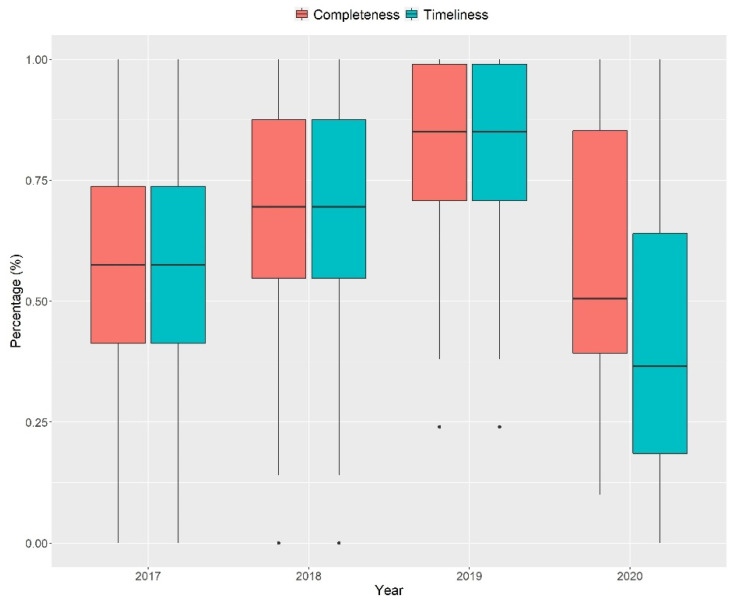
Boxplot of national completeness and timeliness of Indonesia’s EWARS (mean and std. deviation).

The study extracted completeness and timeliness by each year from each district in Indonesia for the years 2017 through 2020. Figure 2 shows national distribution of completeness and timeliness rate. Between 2017 and 2019, there is a consistent improvement in both completeness and timeliness indicator. However, in 2020 some provinces managed to keep the reporting quality, but most of them had fallen and even had worse reporting quality than in 2017.

The study then classified the provinces and districts into six regions based on adjacent locations between each province. Sumatera consists of 10 provinces, Java and Bali consists of 7 provinces, Kalimantan consists of 5 provinces, Nusa Tenggara has 2 provinces, and Maluku and Papua, as the eastern-most part of the country in one region, has 4 provinces. From these data, the research team calculated the mean and standard deviation for each region.

**Figure 3:**
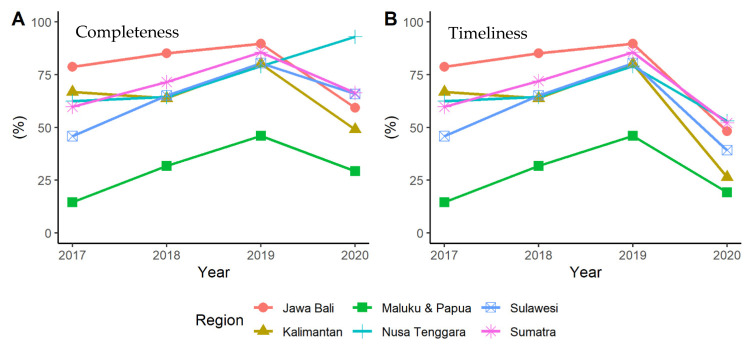
Percentage trends of (**A**) Completeness and (**B**) Timeliness from each region between 2017–2020.

Figure 3 describes the percentage of districts that reached 90% completeness and timelines in each region. All regions experienced significant decreases in both timeliness and completeness in 2020 except for Nusa Tenggara, which showed an increase in completeness in 2020 (Figure 3A,B).

Table 1 shows the mean and standard deviation of the completeness (Table 1a) and timeliness (Table 1b) of reporting from all regions in Indonesia. From 2017 until 2019, we observe a dramatic increase in both completeness and timeliness from all regions. The reporting quality also improves for each year judged from increasing of mean percentage and decreasing of standard deviation, suggesting a reduction in variability from each province. However, in 2020, along with decreasing in report quality, the quality of reports is more dispersed, which signifies inequality of districts’ capacity to respond to COVID-19 while maintaining routine activity reporting. 

The study tested the differences of national completeness and timeliness mean rate between years using non-parametric tests. First, we found significant differences between years using Kruskal–Wallis test for both report completeness and timeliness (*p* < 0.05). Then, the year with more differences used the Pairwise Wilcoxon Signed Rank test which shown in Table 2. In Table 2a, the mean of completeness rate in 2019 has the most significance different from 2017 which means there is a dramatic increase in the completeness rate report. Between 2020 and 2019, there is also a significant decrease in completeness rate (*p*-value ≤ 0.05).

Table 2b shows significant differences between the years 2020 and 2019 signify a notable decrease in report timeliness between those years. However, there is also significant differences between 2019 and 2017, indicating a remarkable increase in report timeliness between those years.

### 3.2. Qualitative Stage (Burden of Disease Surveillance during COVID-19 Pandemic)

In total, we included 43 participants in qualitative data analysis; 38 informants participated in four focus group discussions and five key informants were approached to provide necessary information of the surveillance system. All key informants held postgraduate degrees and had 4–10 years of experience working in disease surveillance activities. Sixty-two percent of the informants were male (*n* = 27) and all of the informants were aged between 28–56 years old. Evaluation of the surveillance system activity at the regional level was based on the CDC’s framework.

#### 3.2.1. System Description

The study assessed whether the present system has sufficient detail to enable stakeholders and other interested parties to comprehend and function within it. According to the in-depth interviews and focus group discussions, the provincial health office acknowledged that the surveillance system is governed under the Ministry of Health Act number 45 2014 about the Health Surveillance Implementation (surveillance of infectious diseases). Data were collected in accordance with the needs of each program. Surveillance data is routinely collected every week for 23 disease syndromes, except for malaria, which requires laboratory confirmation. The data are collected in the Early Warning Alert and Response System (EWARS) which is known in Indonesia as “Sistem Kewaspadaan Dini dan Respon (SKDR)”. Indonesia is actively implementing an early warning system as part of its commitment to comply with IHR standards and contribute to the achievement of global health security. Participants report that, during the COVID-19 pandemic, there were no alterations or changes to the EWARS system (Table 3).

#### 3.2.2. Outbreak Detection

Outbreak detection needed a system to successfully identify an epidemic at the earliest possible stage through complete and timely reported data. EWARS data is collected and reported by staff at the public health center every week via SMS or manual entry into the website. District health offices are well linked to the health facilities in some regions allowing for immediate data collection. On the other hand, some areas with limited staff were unable to input data to the EWARS website.

Detection of other disease outbreaks worsened during COVID-19. One of the focus group discussion participants explained the challenges that occurred at the beginning of the COVID-19 pandemic, where most health services focused on handling COVID-19. Thus, reporting of other diseases, including potential disease outbreaks, diseases that can be prevented by immunization, and other types of diseases, were neglected.

Limited resources resulted in challenges conducting routine surveillance monitoring, particularly for another potential outbreak of disease during the pandemic of COVID-19. Monitoring is carried out in stages, from the center to the province, from the province to the city and district regencies, from the city and districts to the public health center in the field. The response from the provincial health office can be in the form of a weekly bulletin that contains data completeness, accuracy, response to alerts (usually made in the form of a presentation or graphic). If an alert appears on the SKDR, a tiered verification will be carried out. If the alert is correct, then it will be assessed whether the alert caused an outbreak or not; if there is an indication of an outbreak, then staff will proceed to the field and take samples for confirmation in the laboratory (Table 3).

#### 3.2.3. Implementation Challenges

The study describes the challenges affecting the implementation of the EWARS. Human resources are one of the obstacles that all participants raised at both sub-national and national levels. Due to the general staff shortage, surveillance officers may be assigned various responsibilities at public health centers, resulting in increased workload and decreased reporting time. Roles of surveillance staff in health centers are also unclear with uneven distribution. During the pandemic, health surveillance staff were assigned to conduct COVID-19 surveillance, including tracing and daily COVID-19 reporting. Apart from quantity, the quality of surveillance staff is also a problem. There is surveillance personnel with non-surveillance-related competencies, which complicates the understanding of operational definitions and analytical responsibilities. Frequent staff transfers can act as a hindrance, as new staff must re-adapt to their new roles.

Informants from the FGD stated that the presence of COVID-19 influenced their performance in the field. At the beginning of the pandemic, there were districts that did not provide any routine surveillance report, causing lapses in the reporting. Therefore, public health centers capacity is stretched as the outbreak has progressed. They are burdened by limited personnel resources, as most of their time has been dedicated handling and reporting COVID-19 cases. One informant stated that she used to prepare bulletins on a monthly basis, but currently she did not have sufficient time to do so.

In the process of collecting data on the SKDR website, public health center staff who send SMS may not receive feedback, so they must open the website to ensure that reports have been entered on the website (Table 3).

#### 3.2.4. Improvement Strategy

The improvement strategy should be evaluated with the stakeholders and need to be adjusted accordingly. The recommendation should address the possibility of technical implementation. Therefore, the surveillance system could effectively achieve the goal. Human resources are crucial in the data collection and reporting process. One informant recommended appropriate human resources, as well as individuals with the necessary background for their surveillance profession. A suitable background will help them handle their daily responsibilities easier. Both subnational and national staff expressed hope for an increase in the capacity of surveillance staff in the districts.

Apart from adequate human resources, the informants also stated that fewer activities and tasks be performed by public health staff. Therefore, they hope that there will be improvement in terms of the system too. They hope there will be data integration between EWARS and public health care and laboratories that can help in carrying out an outbreak investigation. With this integration, suspected outbreaks can be confirmed quickly, with immediate follow-up (Table 3).

## 4. Discussion

In this study, the EWARS implementation was assessed in Indonesia through quantitative and qualitative methods based on a framework developed by CDC [30]. The quantitative analysis showed an overall national completeness and timeliness, showing improvement between 2017 until 2019. However, both metrics fell significantly in 2020 in all regions except Nusa Tenggara, where completeness increased. Statistical analysis shows significant differences of both metrics between 2020 and 2019 indicating falls of both metrics. The study also found a decrease in the national reporting quality decreases in 2017. In addition, results showed a clear deleterious impact of the pandemic and on routine surveillance activity at the PHC level.

The complementary qualitative analysis adds several findings to the subsequent analysis. First, regional public health officials could describe clearly the function of EWARS application. They described it as a mandatory weekly aggregated data reporting through the application regarding potentially outbreak and vaccine-preventable diseases. The application will give a warning when there is a notable increase in the number of cases at healthcare facility level that could lead to outbreak. Then, related healthcare facilities form a team to investigate the alert as response to the potential outbreak signal. To simplify and speed up the investigation, online groups are formed using popular mobile messenger apps such as WhatsApp.

Some participants mentioned the limitation of surveillance human resource in PHC. This human resource limitation is mostly notable in rural eastern Indonesia—Maluku and Papua. According to the participants, surveillance officers in PHCs have a high turnover rate compared to other staff, leading to a lack of continuity in program support and knowledge transfer. It was mentioned that human resources in PHC were limited and there were some areas that were not evenly distributed. Moreover, as described in quantitative analysis, the pandemic of COVID-19 has, since early 2020, interfered the routine surveillance reporting activity. The COVID-19 pandemic in 2020 has worsened the situation because the human resources at PHC are allocated to handling and collecting and reporting COVID-19 cases every day.

The burden of COVID-19 increases their workload. Routine surveillance activity are now added with COVID-19 active surveillance due to political pressure from the national and regional government. Due to the limitation of human resource, the management of PHC have no choice to shift the surveillance staff to focus on COVID-19 surveillance activity.

Qualitative analysis also indicates significant gaps in infrastructure. EWARS application already offers several alternative reporting methods such as via text messages and WhatsApp; however, a stable mobile network is required to perform such tasks.

From the side of EWARS users, there are some technical problems that make this system not yet user friendly. The PHC information system has not been integrated with EWARS, therefore there is repeated data entry of the same data by staff at the PHC. This system also does not provide feedback for those who have collected data, more specifically via SMS. This requires staff to check the EWARS website regularly. Additionally, there is no reminder if the staff at the PHC have not reported. All of these factors serve to reduce motivation to input data in a timely manner.

The study brings an update to the previous study by Hapsari et al. in four Indonesia regions. Our research findings show that the western regions maintains a quality of reporting data year by year since the first enactment of EWARS [22]. However, our findings also confirm limitation experienced by eastern regions (Maluku and Papua) that still has not been solved, especially in providing epidemiologists or particular staffs focused on disease surveillance [23]. 

Limited numbers of trained surveillance officers and infrastructure gaps between regions hinder the national surveillance program. The gaps in reporting quality may produce unreliable and inconsistent data. To overcome the challenges, several recommendations can be offered. Public health threats can occur at any time. A reliable EWARS should withstand public health challenges by reducing dependence on human resources. Given the proper implementation of automated technologies, we expect human resources to be more efficiently used [31]. Several countries with a well-established national e-Health system could build an automated EWARS architecture based on a wide network of electronic medical record systems. The system has been proven to work in respiratory syndrome use cases [32,33]. Other countries with single-payer national health insurance system may use its potential to support infectious disease surveillance [34]. In particular, it would be useful to integrate EWARS with the National Health Insurance (NHI) data.

In 2014, Indonesia established a National Health Insurance (NHI) scheme operated by Health Social Insurance Administration Agency (BPJSK). In 2020, the NHI scheme already covered around 90% of the Indonesian population and has proven to increase healthcare access [35]. With its large participation rate and extended information system, including public and private clinics and hospitals, NHI provides more complete data than EWARS. Data integration between EWARS and the NHI database would leverage complementary data sources for improved disease surveillance [34].

The high turnover rate of PHC surveillance staff, inconsistent handover to new staff and the lack of training for newly appointed surveillance staff also interferes with the EWARS reporting process. E-learning may serve to increase self-efficacy [36,37], knowledge outcomes [38,39,40], instructional design [41], and user satisfaction [42,43]. E-learning platforms allow surveillance staff to have updated information on EWARS reporting and ways to overcome challenges in data completeness and timeliness. 

This study used available public data from 2017–2020. While a longer time series might have yielded a better understanding of trends, the system was not operating in all provinces before 2017. Due to travel restrictions, data collection was conducted online. The distraction of incessant demands of COVID reporting was evident in our respondents during FGDs and in-depth interviews.

The main limitation of this research is the limited number of participants who come from epidemiology personnel in the area who can take part in the discussion because, at the same time, several health resources are intensifying contact tracing for COVID-19. The discussion was conducted online in each province so that it was possible for participants not to convey all the problems they faced. However, the researcher has retyped and sent back the input from the participants so that they can be checked again for the correctness of the data. To maximize research data collection, it should be undertaken face-to-face and directly see the current condition of the implementation of the EWARS.

## 5. Conclusions

Our study evaluated the quality of EWARS implementation in Indonesia. The results show existing gaps in surveillance reporting quality based on EWARS application in Indonesia and the burden of disease among surveillance worker in primary health care facility during COVID-19 pandemic. Gaps were found in human resources and infrastructure aggravated by the COVID-19 epidemic. Poorly distributed human resources in surveillance affects both the completeness and timeliness of EWARS reporting. Moreover, the shifting of workload to focus on COVID-19 influenced the report quality in most regions. It is recommended that a public servant recruitment reformation be established to distribute the epidemiology human resources evenly between region. E-learning platform may be built to cope with high turnover in epidemiology resources in PHCs. In addition, an automated reporting process may ameliorate the human resource and infrastructure shortcoming in rural areas. EWARS could be integrated with various data resources that automatically collects data directly from electronic health record in healthcare facilities, laboratories and NHI claims database.

## Figures and Tables

**Table 1:** (**a**) Percentage of completeness reports between 2017–2020 in Indonesia. (**b**) Percentage of timeliness reports between 2017–2020 in Indonesia.

(a)				
Region	Completeness (%)			
	2017	2018	2019	2020
Sumatra	59.86 ± 22.5	71.60 ± 24.9	85.75 ± 19.9	66.36 ± 30.7
Java and Bali	78.77 ± 21.8	85.18 ± 15.0	89.67 ± 10.5	59.33 ± 28.0
Kalimantan	66.81 ± 16.3	63.85 ± 17.4	80.07 ± 18.4	48.99 ± 15.5
Sulawesi	46.03 ± 14.7	65.15 ± 13.0	80.52 ± 11.5	65.93 ± 29.0
Nusa Tenggara	62.27 ± 10.9	64.55 ± 7.71	79.09 ± 15.4	93.18 ± 9.6
Maluku and Papua	14.73 ± 20.9	31.63 ± 32.0	46.10 ± 24.8	29.00 ± 19.0
**(b)**				
**Region**	**Timeliness (%)**			
	2017	2018	2019	2020
Sumatra	59.86 ± 22.5	72.04 ± 24.1	85.75 ± 19.9	52.04 ± 34.1
Java and Bali	78.77 ± 21.8	85.18 ± 15.0	89.67 ± 10.5	48.15 ± 29.6
Kalimantan	66.81 ± 16.3	63.85 ± 17.4	80.07 ± 18.4	26.37 ± 20.1
Sulawesi	46.03 ± 14.7	65.15 ± 13.0	80.52 ± 11.5	38.94 ± 30.3
Nusa Tenggara	62.27 ± 10.9	64.55 ± 7.7	79.09 ± 15.4	53.18 ± 23.7
Maluku and Papua	14.73 ± 20.9	31.63 ± 32.0	46.10 ± 24.8	19.21 ± 24.3

**Table 2:** (**a**) Pairwise Wilcoxon Signed Rank result of national report completeness mean rate compared between years. (**b**) Pairwise Wilcoxon Signed Rank test result of national report timeliness rate compared between years.

(a)			
Year	2017	2018	2019
2018	0.28		
2019	<0.001 *	0.08	
2020	0.98	0.38	0.03 *
**(b)**			
**Year**	**2017**	**2018**	**2019**
2018	0.09		
2019	<0.001 *	0.05	
2020	0.05	<0.001 *	<0.001 *

* *p*-value < 0.05.

**Table 3:** Summary of qualitative analysis on EWARS system in Indonesia.

Themes	Sub-Themes	Selected Quote
System Description	Mandatory program at all health office	“*Surveillance are a critical component of all Ministry of Health programs; this implies that each department within the health office must have a surveillance program.*”
	Surveillance on potentially outbreak and vaccine preventable diseases	“*We actively 23 monitored disease with the potential outbreak through SKDR program including vaccine preventable disease.*”
	Alert and response	“*When we got alert in SKDR, we immediately establish an investigation team and begin searching for further instances in the area.*”
Outbreak Detection	Aggregate data reporting	“*We send the data in aggregate form, when there is data that match the definition for possible outbreaks, an alert will automatically appear.*”
	Online collaboration	“*We have SKDR whatsapp group in the national level to provide a feedback every week to the province.*”
Implementation Challenges	Human resource limitation	“*The provincial health office has a limited human resource available for the surveillance team. As a result, various responsibilities were assigned to the same staff*”
		“*Human resources are not evenly distributed in almost all public health centers.*”
	Increasing workload	“*It seems that the district staff are also a bit overwhelmed to have to handle the surveillance, which includes SKDR, STP (“integrated disease surveillance”) and COVID.*”
		“*There is a pandemic so many staff are focused on dealing with this COVID pandemic so yeah.*”
		“*Sometimes the problem we face is the overload in the system due to a lot of text messages. The second problem is that the staff at the Public Health Center do not receive a response from the SMS center whether they are texting with the right format or not.*”
	Management shifting	“*There is no changes in the EWARS system during pandemic, however we need to stop the report and focus to the management of COVID.*”
		“*Staff at the public health center and at the hospital stopped reporting because they only focused on dealing with COVID-19.*”
Improvement Strategy	Additional epidemiologists at primary health care level	“*We hope that they have a background on disease surveillance, so that it is not too difficult for us to increase their capacity.*”
		“*It is necessary to strengthen competency and trained Surveillance personnel. It is also necessary to add a trained surveillance team at the Provincial and Dis-trict/City Health Offices.*”
	Interoperability with laboratory	“*I hope SKDR is not only limited to monitoring for suspects but also being able to link with the laboratory…*”

## Data Availability

The data presented in this study are available on request from the corresponding author. The data are not publicly available due to sensitive information contained and because transcripts were made only in Indonesian language.

## Supplementary Materials

The following supporting information can be downloaded at: https://www.mdpi.com/article/10.3390/ijerph19052728/s1, Supplement 1. Informed consent sheet; Supplement 2. Interview Guidelines; Supplement 3. Detailed Province Reporting Quality (Completeness & Timeliness).
