# Supplementary material for: Quality of National Disease Surveillance Reporting before and during COVID-19: A Mixed-Method Study in Indonesia
Source: Int J Environ Res Public Health. 2022 Feb 26;19(5):2728. doi: 10.3390/ijerph19052728 (PMC8910184; doi:10.3390/ijerph19052728)

Supplement 1. Informed consent sheet

Explanation Sheet

Hello, my name is (researcher/enumerator name) will conduct a research entitled "Quality of National Disease Surveillance Reporting Before and After COVID-19: A Mixed-method study in Indonesia" included in the INSPIRASI Project (Improving quality of disease preparedness, surveillance and response in Indonesia). This research was sponsored by the Centers for Disease Control (CDC) in collaboration with Center of Health Policy and Management Faculty of Medicine, Public Health, and Nursing, Universitas Gadjah Mada (CHPM FKKMK UGM)

This research aims to:

1. Assess national disease surveillance reporting procedures from early detection, registration, confirmation, reporting, analysis, and dissemination the feedback to the public before and after COVID-19
2. Assess what need to improve in surveillance information systems
3. Assessing PHEOC readiness at the provincial level in dealing with epidemics or pandemics

The research team invited you to participate in this research. This research requires about 15 panelists, with a period of 1 to 1.5 hours and will be conducted 1 in-depth interview or 1 Focus Group Discussion (FGD).

A. Volunteering to participate in research

You are free to choose to participate in this research without coercion. If you have decided to participate, you are also free to resign/change your mind at any time without being subject to any fines or sanctions.

B. Research procedure

If you are willing to participate in this research, you are required to sign this double consent sheet, one for you to keep, and one for the researcher. The next procedure is:

1. You will be interviewed by the research team to ask questions that have been in the research instrument.
2. The interview will be conducted for 1 to 1.5 hours.
3. Interviews are conducted once.
4. The researcher will record during the in-dept interview and/or FGD process.
5. The researcher will make a transcript of the interview.

C. Obligations of research subjects

As a research subject, you are obliged to follow the rules or research instructions as written above. If there is anything unclear, you can ask the researcher further.

D. Risks and Side Effects

The research will describe your knowledge about the implementation of national disease surveillance reporting before and after COVID-19. The risk for the subject could be data leakage. To prevent data leakage and maintain the confidentiality of the subject's identity, each subject will be given a data source code and in the publication of research results will be published without the identity of the research subject.

E. Benefits

Formulate gaps of national disease surveillance reporting before and after COVID-19 to improve quality of the disease surveillance system in Indonesia.

F. Confidentiality

All information relating to the identity of the research subject will be kept confidential and will only be known by researcher and the research staff. The results of the research will be published without the identity of the research subjects.

G. Compensation

Participants will receive compensation one hundred and fifty thousand rupiah.

H. Financing

All research-related costs will be borne by researchers and sponsors.

I. Additional Information

You can ask all the things that are not clear about this research. If you need an explanation, you can contact dr. M. Hardhantyo, MPH, Ph.D. at (phone number) at the Center of Health Policy and Management Faculty of Medicine, Public Health, and Nursing Universitas Gadjah Mada (CHPM FKKMK UGM), North Sekip, Yogyakarta 55281, Indonesia.

You can also ask about research to the Medical and Health Research Ethics Committee of Faculty of Medicine UGM (Phone. 0274-588688 ext 17225 or +62811-2666-869; email: mhrec_fmugm@ugm.ac.id).

Statement of Informed Consent

I, voluntarily participated in this research launched by the Centers for Disease Control (CDC), as well as CHPM FKKMK UGM, under Dr. dr. Hanevi Djasri, MARS, FISQua, as the Head of INSPIRASI research. I understand that this research was designed to gather expert perspectives on national disease surveillance reporting before and after COVID-19, of 15 panelists.

1. My participation in this project is voluntary. I can withdraw and stop participation at any time without penalty.
2. If I feel uncomfortable with anything during the interview session, I reserve the right to refuse to answer questions or end the interview.
3. The interview will last 1 to 1.5 hours and will be conducted once. The researcher will write a note during the interview. The interview will be recorded audio and some pieces of the conversation will be taken for reporting. If I didn't want to be recorded, I wouldn't be able to participate in the research.
4. I understand that the researcher will not identify my name in any report using the information obtained from this interview, and my confidentiality as a participant in this research will be maintained.
5. I understand that the use of records and data will then be processed for further analysis in accordance with data use policies that protect the anonymity of individuals and institutions.
6. I understand that this research has been reviewed and approved by the Medical and Health Research Ethics Committee of Faculty of Medicine, Public Health and Nursing, Universitas Gadjah Mada (protocol code KE/FK/0682/EC/2020)
7. I have read and understood the explanation given to me. I have got answers to all my questions, and I voluntarily agreed to participate in this research.
8. I have been given a copy of this consent form.

By signing this form, I agreed to participate in this research.

Subject signature: Date __ / __ / ____

Clear Name:

Witness signature:

Full Name :

General Information (Filled out by interviewer)

1. Interviewer :
2. Minutes :
3. Date :
4. Time : Start ____________and Finish __________
5. Location :
6. Use

   Voice Recorder : (Yes/No), If Not, the reason:

Characteristics of Informants (Filled by interviewer)

7. Name : _______________________

8. Age : ________________________
9. Gender : ________________________
10. Education : ___________________________
11. Position : _______________________

12. Previous position : _______________________
13. Length of time serving current position : _________________

Informant code : _________________

<u>Supplement 2. Interview Guidelines</u>

Procedures for opening an interview

Good morning/ noon/ afternoon, let me introduce us, I am … and my partner… . Thank you for taking the time. The purpose of this activity is that we want to learn from you about your experience in the disease surveillance program implementation. We will ask some questions, and please answered freely expressing the opinion. There are no right or wrong answers. This interview will last from 1 hour to 1.5 hour. To facilitate transcription, this interview will be recorded. We will keep all the information; no names and titles will be released. Do you agree to have this interview recorded?

Do you have any questions before we start?

Issue(s) explored	Question	Probbing/ Dunning
System description	Can you tell us about surveillance system in Indonesia?	What are the programs? What is the background of the program? What does the program refer to?<br><br>How about disease surveillance system?
Outbreak detection	How is the implementation of outbreak detection from EWARS data?	How is the current process for EWARS data collection?<br><br>How is the reporting process? What data are reported and how is the process?<br><br>How is the Provincial Health Office monitors and evaluates EWARS? How about the Ministry of Health do it? Is there any verification of data from which the report is submitted?<br><br>Please tell us about the process of data analysis and data dissemination to the public?

Implementation challenges	What are the obstacles in EWARS implementation?<br><br>How is the EWARS implementation during COVID-19?	What are the obstacles in EWARS implementation in Public Health Center?<br><br>How is the data entry process in EWARS? Are there any obstacles during the data collection process?<br><br>What data are reported and how is the process? Is there a specific schedule for reporting? What about the EWARS report indicators so far?<br>Are there any obstacles when reporting data?<br><br>What about the allocation of human resources related to EWARS so far? Are there any problems with it during COVID-19?
Improvement strategies	What improvement strategies have been implemented for EWARS?	What is your organization do to support EWARS?<br><br>What are the suggestions for improving EWARS?

Supplement 3. Detailed Province Reporting Quality (Completeness & Timeliness)

Jawa & Bali

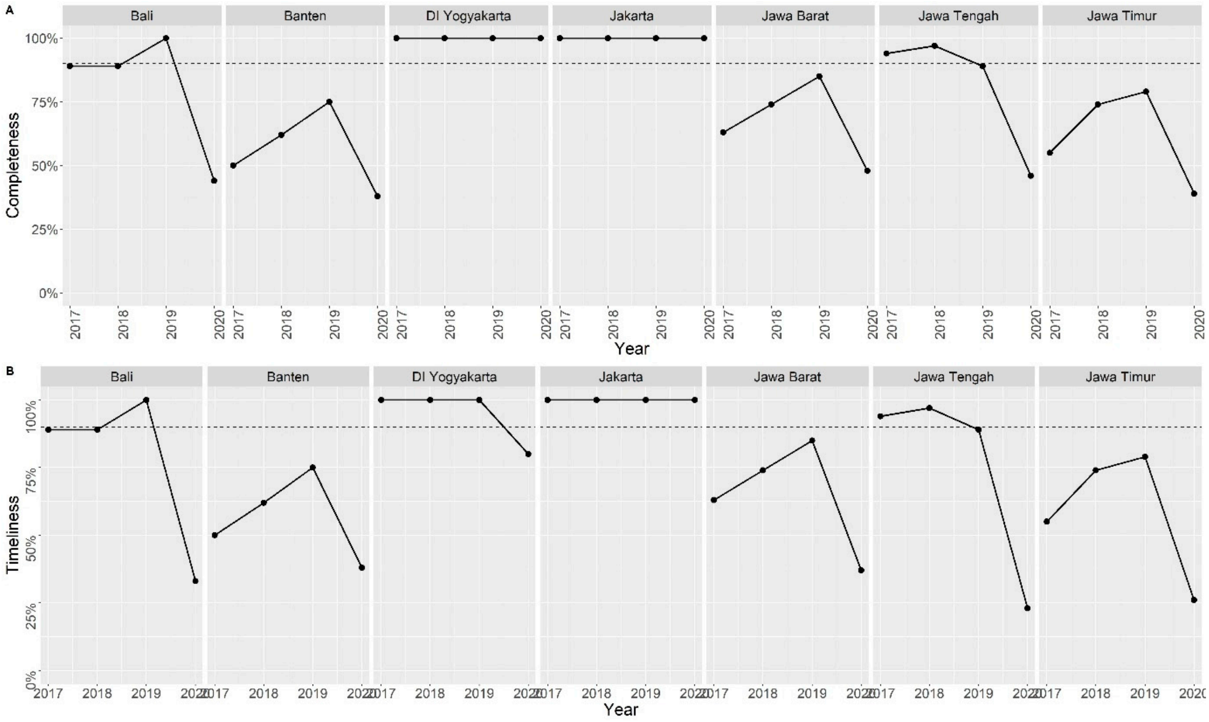

Sumatera

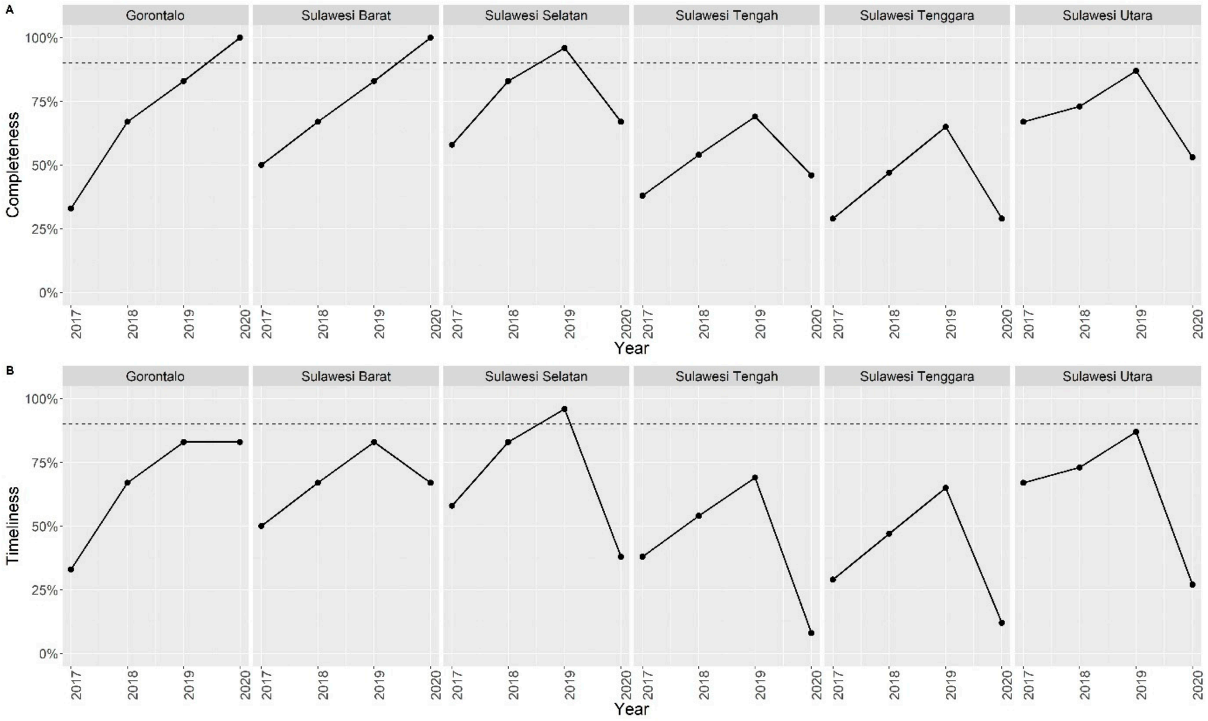

Kalimantan

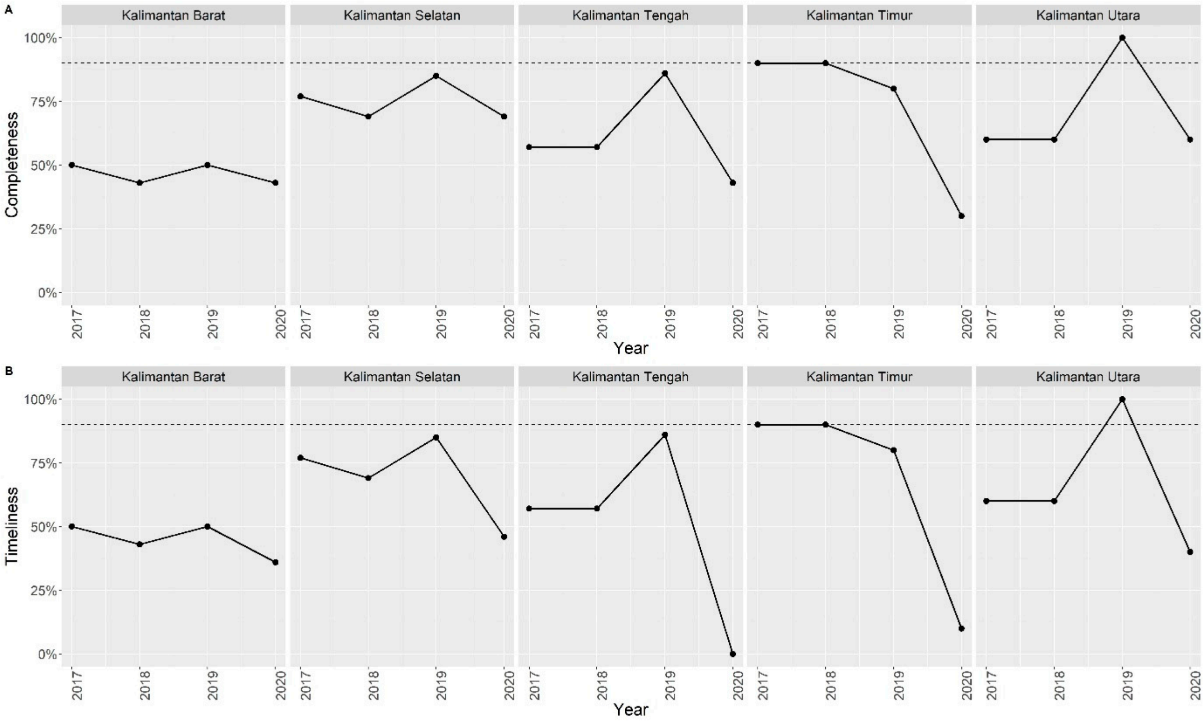

Nusa Tenggara

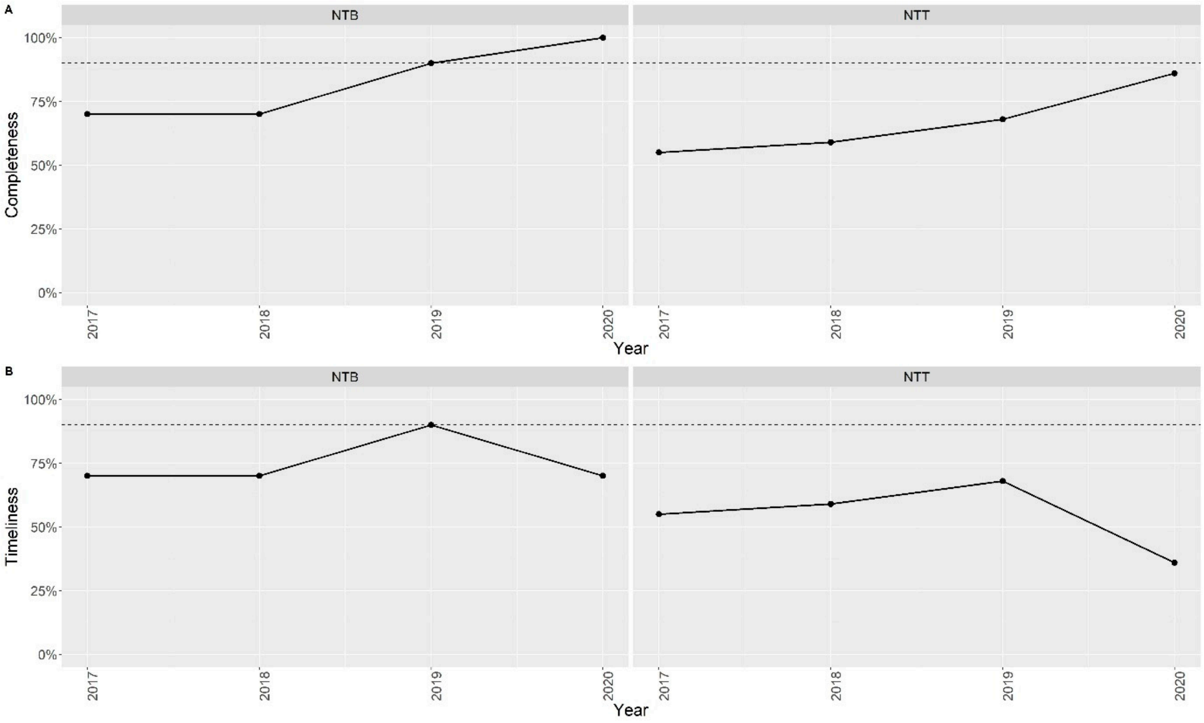

Maluku & Papua

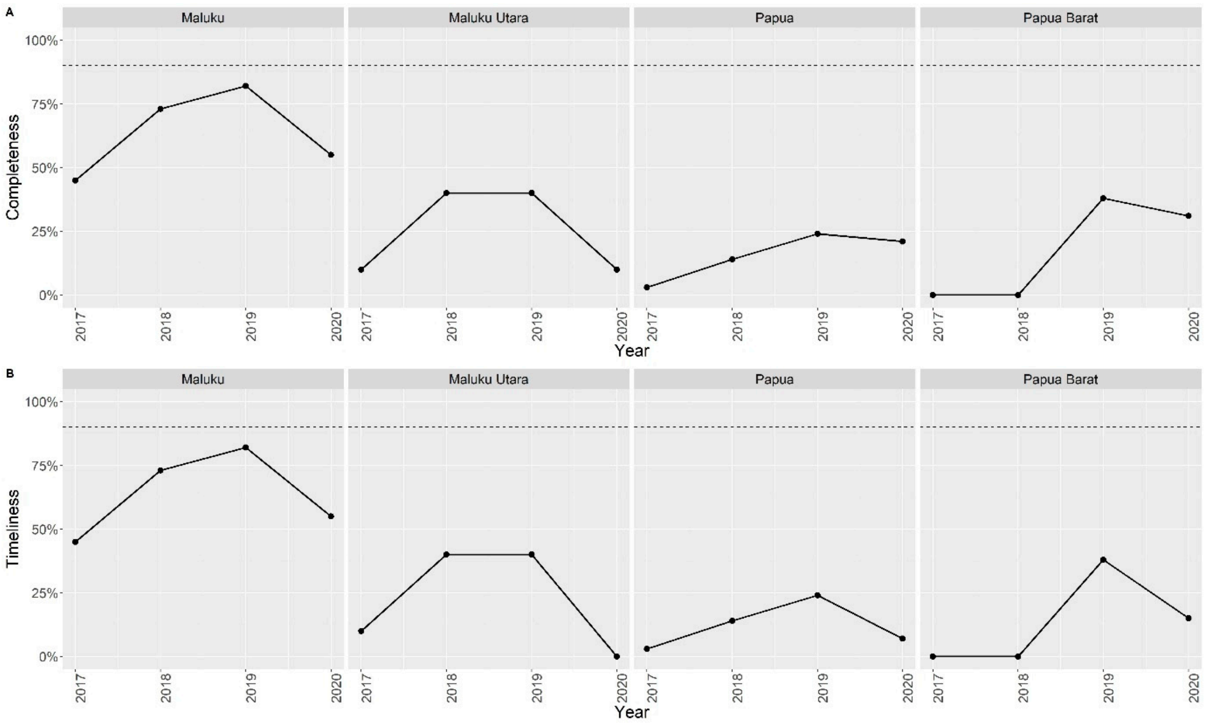

Supplement: Supplementary file 1 [file ijerph-19-02728-s001.zip › ijerph-1589592-supplementary.pdf]
